# Water provisioning increases caged worker bee lifespan and caged worker bees are living half as long as observed 50 years ago

**DOI:** 10.1038/s41598-022-21401-2

**Published:** 2022-11-14

**Authors:** Anthony Nearman, Dennis vanEngelsdorp

**Affiliations:** University of Maryland, College Park, MD USA

**Keywords:** Animal disease models, Ageing, Disease model, Diagnostic markers, Predictive markers

## Abstract

The high loss rates of honey bee colonies drive research for solutions aimed to mitigate these losses. While honey bee colonies are superorganisms, experiments that measure the response to stressors often use caged individuals to allow for inference in a controlled setting. In an initial experiment, we showed that caged honey bees provisioned with various types of water (deionized, 1%NaCl in deionized, or tap) have greater median lifespans than those that did not. While researching the history of water provisioning in cage studies, we observed that the median lifespan of caged honey bees has been declining in the US since the 1970’s, from an average of 34.3 days to 17.7 days. In response to this, we again turned to historical record and found a relationship between this trend and a decline in the average amount of honey produced per colony per year in the US over the last 5 decades. To understand the relationship between individual bee lifespan and colony success we used an established honey bee population model (BEEHAVE) to simulate the predicted effects of decreased worker lifespans. Declines in downstream measures of colony population, overall honey production, and colony lifespan resulted from reduced worker bee lifespans. Modeled colony lifespans allowed us to estimate colony loss rates in a beekeeping operation where lost colonies are replaced annually. Resulting loss rates were reflective of what beekeepers’ experience today, which suggests the average lifespan of individual bees plays an important role in colony success.

## Introduction

Laboratory cage studies that measure the median lifespan of adult worker honey bees are central to studying the etiology of honey bee diseases^1–3^, the effects of honey bee health products^4^, and the risk associated with single and combined exposure to pesticides and other risk factors^5,6^. Cage studies have advantages over colony level trials, as they help standardize or control for environmental variables. They are also inexpensive with rapid turnaround and allow for more precise quantification of physiological responses to applied exposures.

The standardized protocol for cage studies detail many variables known to effect cage trial outcomes—such as temperature, humidity, and diet^2^. These protocols do not, however, contain empirically derived recommendations regarding water supplementation during trials. Rather, the recommendation states that 50% carbohydrate solutions are sufficient for hydration, and so no water is needed unless the carbohydrate source is solid. This rational ignores the complex physiological mechanisms and behavioral responses that animals have to water^7,8^. In addition to individual osmoregulation, honey bees’ forage for water in order to collect micronutrients^9,10^, dilute food concentration for feeding brood, and regulate colony and individual temperature^11,12^. Altogether this suggests water is a dietary need, and its absence would create artificial and unneeded stress to caged bees.

An optimal cage environment and diet, which minimizes stress, contribute to study conditions that are better reflective of life in the colony, and so ensuring that study results are more reflective of real-world scenarios. Common measured outcomes in cage studies are median lifespan (50% population loss) and longevity (average lifespan). Worker bee longevity has direct effects on colony productivity—the longer a worker bee lives, the longer it can forage, and the more honey a colony produces^13,14^. Population models that detail the intricacies of colony population dynamics find that increases in adult bee mortality or changes to the overall age structure can predict colony death^15–20^. Much of our understanding of general honey bee longevity, however, is based on the work of early bee scientists, who individually tagged bees and returned them to their colonies for observation^21–23^. As current assertions are built on knowledge obtained in the 1950’s, prior to decades of exposure to factors thought to drive today’s colony loss rates, some reassessment of our baseline knowledge may be necessary.

Considering the biological importance of water and the commonality of lifespan as a measured outcome, the lack of studies comparing the two is somewhat surprising. We set out to correct this, hypothesizing that water provisioned caged bees would have longer median lifespans than control bees. We did, in fact, find that caged bees with access to water had longer median lifespans than those that did not. When comparing the resulting median lifespans to that of bees in cage trials performed in the 1970’s, our control bees lived half as long. This inspired a much deeper review of the cage trial literature, resulting in a dataset that permitted us to identify diets and other factors that associated with changes in honey bee median lifespan, as measured by the control groups in those studies. The results of this analysis suggested a reduction in the median lifespan of caged bees over the past five decades. We then used existing honey bee population models to predict the impact possible changes in adult worker bee lifespan would have on colony productivity and survival, and then compared these to real world data. We hypothesized that decreased worker bee lifespan should correlate to decreased honey production, as measured per colony, and should increase colony loss rates.

## Results

**Figure 1:**
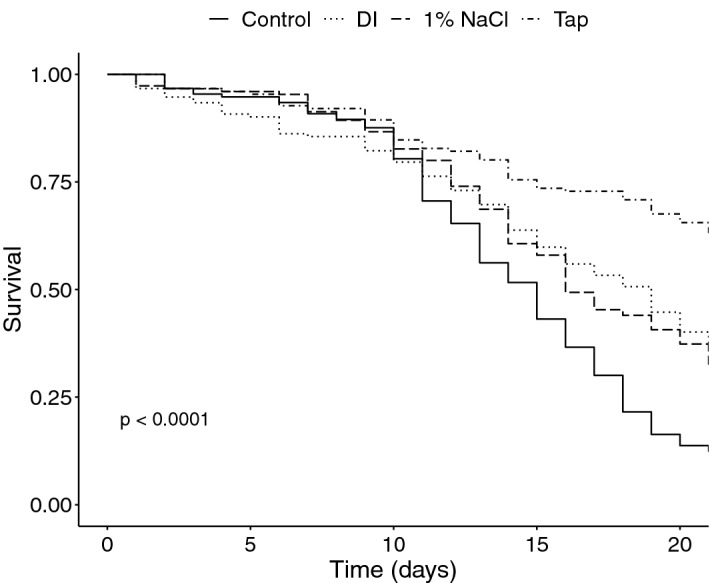
Kaplan–Meier survivorship curves showing Survival Probability between cages of bees offered 50% sugar syrup, pollen substitute, and either no water (Control), tap water (Tap), deionized (DI) water, and a 1% NaCl deionized water solution.

**Figure 2:**
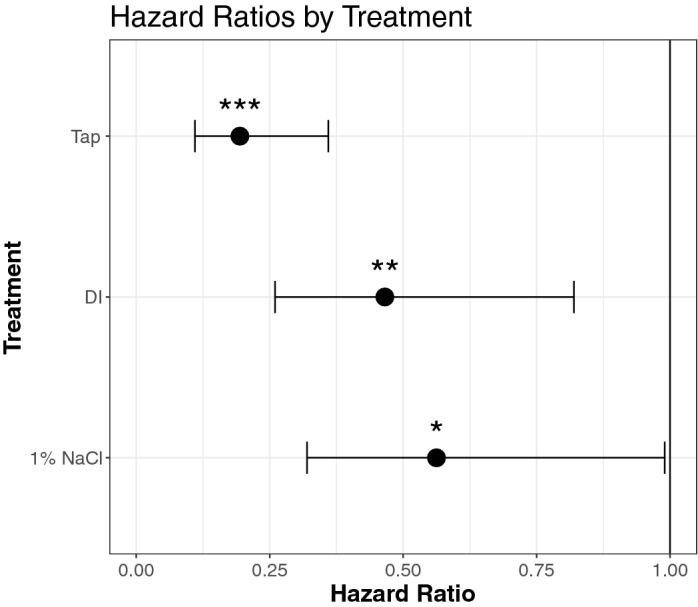
Cox Proportional Hazards Ratios for cages of bees given supplemental water (either tap water, deionized (DI) water, or a 1% NaCl solution in deionized water), and a control group offered no supplemental water. Whiskers represent the 95% confidence intervals, none of which cross the null hypothesis of HR = 1 (Vertical line) demonstrating the reduced hazard of water provisioning in any form. * α < 0.05, **α < 0.01, ***α < *0.001.*

**Figure 3:**
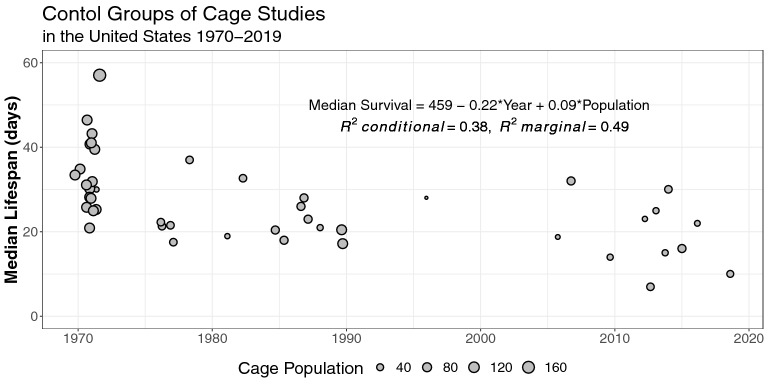
Median lifespans for the control groups of cage studies performed in the US between 1970 and 2019. Significant differences in median lifespans were negatively correlated with time and positively correlated with cage populations. Point size represents cage population. Equation represents the mixed modeling results.

**Figure 4:**
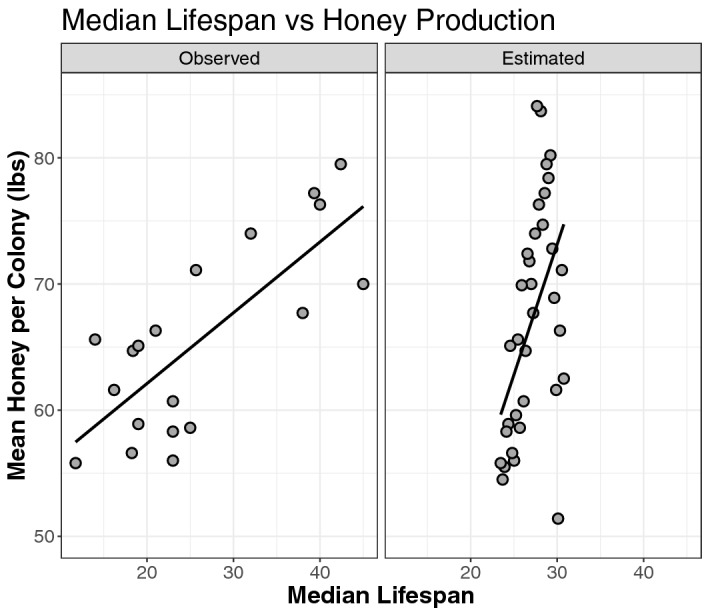
Median lifespan of caged bees, either mean observed or model estimated per year vs mean honey produced per colony in the US 1987–2019.

**Figure 5:**
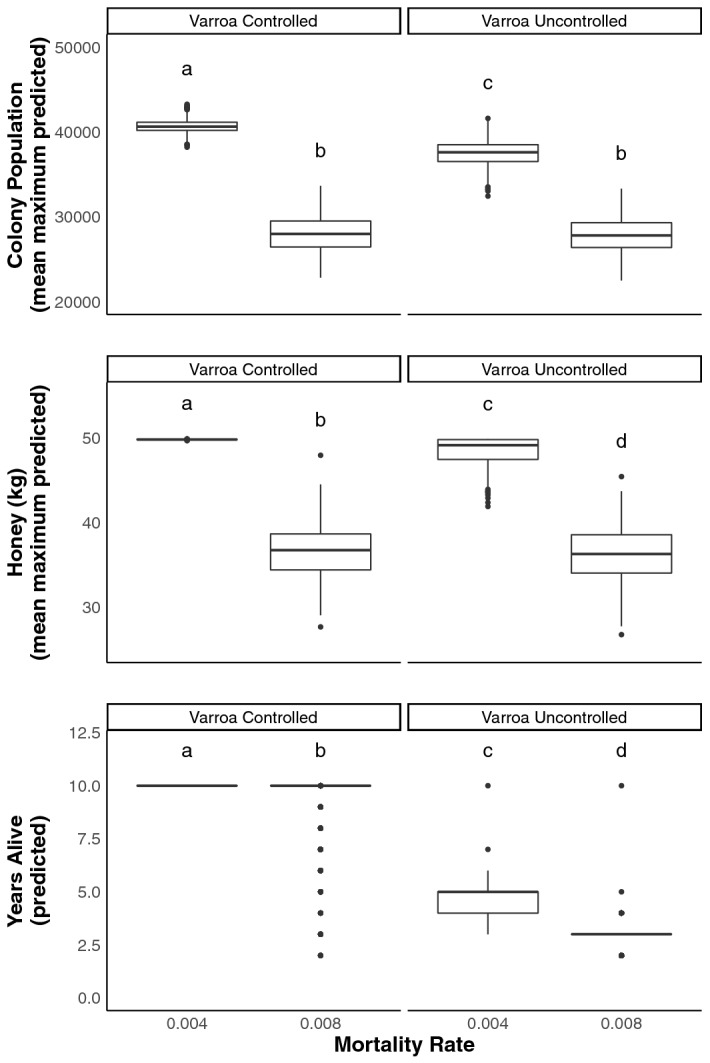
BEEHAVE modelling results for different in-hive mortality rates and Varroa control regimes. Each box represents 1000 replicates. Daily worker bee in-hive mortality rate was left to either the default setting of 0.004 (mid twentieth century estimated lifespan) or increased to 0.008 (2010–2019 estimated lifespan). Models employing each mortality rate were repeated with the adjustment of Varroa treatment. All other model parameters were left to default setting. Letters indicate pairwise comparisons using a Wilcoxon Rank Sum test with the Benjamini–Hochberg procedure for controlling false discovery rate.

Our cage study reveals that offering supplemental water to bees in cages increases their median lifespan. The control group, receiving no water, had a median lifespan of 15 days, while bees provisioned with tap water had a median lifespan of greater than 21 days, the end of the experiment, where those cages retained 67% of their population (Figs. 1 and 2). Our literature analysis on the median lifespans of historical cage studies (n = 46) shows a positive correlation with the cage populations and median lifespans for the control groups of those publications (Fig. 3). This analysis also showed a clear decrease in the median lifespan of control bees in studies conducted in the US over the last 5 decades (Fig. 3). Considering the importance of worker bee lifespan on colony productivity^13^, we hypothesized that if such a relationship were real we would see a correlation between median lifespan and historic honey production figures. We used median lifespan over average lifespan in our literature analysis because there were more studies that included the former (46 vs 2). We found declining median life spans, both observed and modeled, correlated to decreased honey produced per colony in the US. (Fig. 4). Our *in-silico* population modeling also demonstrates the negative effect these shortened median lifespans can have on theoretical honey yields and colony survival (Fig. 5, Supplementary Table S1). When current-day bee lifespans and *Varroa* impacts are used in the model, it predicts decreases in colony survival that, when extrapolated to the operational level, predict cyclical operational losses that fit well within the range estimated inUS beekeeping operations over the last 14 years (Supplementary Fig. S1)^1^.

### Cage study

*Bees that have access to water are more likely to survive more than 20 days than those that do not.* Kaplan–Meier survivorship curves show that the survival probability of bees offered either tap water, deionized water, or a 1% NaCl deionized water solution is greater than bees offered sugar syrup only (*p* < 0.0001, Fig. 1). For the bees offered no water (Control), the probability of surviving 21 days is 12.5%, whereas the bees offered deionized water, with or without 1% NaCl, is ~ 37.5%, and is 67% for bees offered tap water (Fig. 1).

To account for possible effects of colony and cage of origin, we calculated the Cox Proportional Hazard Ratios (HR) using a mixed effect model. Bees in the control group experienced a relative overall death rate 5 times higher than those with access to tap water (HR = 0.19, *p* < 0.0001). Similarly, bees in the control group died roughly twice as fast as those offered deionized water (HR = 0.47, *p* < 0.0086) or a 1% NaCl deionized water solution (HR = 0.56, *p* < 0.046) (Fig. 2).

### Literature analysis

Our literature search identified 111 cage studies published between 1970 and 2019. Criterion for inclusion in the analysis was limited to studies that reported median lifespan as an experimental outcome, newly emerged bees as the source specimen, and the experimental design variables related to cage environment. Due to the lack of consistency of data reported from countries other than the United States over the period, we removed non-US studies leaving us with 68 trials reported in 26 publications (see Supplementary Table S2). The average median lifespan of caged bees in the 1970’s was 34.3 (± 1.5) days and has reduced to 17.7 (± 2.0) days 50 years later (Table 1). Our linear modeling of median lifespan included the variables cage starting population and diet (water, pollen, and/or pollen substitute provided). Other variables were excluded from our model because of lack of reporting or diversity in methodology. These included: the season bees were harvested (n = 11), the use of dietary honey (n = 3), sugar syrup concentration (88% of studies used 50% sugar syrup), when water was included in the design (n = 50), the type of water used was reported infrequently (n = 7). In order to eliminate within-study effect sizes of modeled variables, only the most basic control group experimental designs were included in the regression. The resulting parsimonious model of 46 trials across 25 publications suggests the median lifespan of US worker bees has declined at a rate of 0.22 days per year since 1970 (SE = 0.090, *t-value* = − 2.450, *Pr(* >*|t|)* = 0.021, Fig. 3). Also, each bee in a cage’s population is associated with a 9.3% increase in median lifespan (SE = 0.043, *t-value* = 2.147, *Pr(* >*|t|)* = 0.040, Fig. 3).

**Table 1:** Summary of experimental design variables and median lifespans by decade for all control groups of survivorship cage trials conducted in the US from 1970 to 2019.

Decade		Population			Water	Pollen	Pollen substitute	Median lifespan
	n	Mean (SE)	Median	Range	n	n	n	Mean (SE)
1970	42	68.57 (5.91)	75	10–170	34	25	23	34.30 (1.51)
1980	8	50 (5.67)	60	20–60	7	2	1	29.71 (4.67)
1990	4	77.5 (22.5)	100	10–100	3	3	1	19.15 (3.56)
2000	2	38 (22)	38	16–60	1	1	0	25.38 (6.62)
2010	12	38.33 (4)	35	20–60	5	6	1	17.67 (2.02)

### Honey production

Published experiments that recorded worker bee median lifespan were reported in 19 of the 32 years for which honey production data was collected by the National Agricultural Statistical Service (NASS) using current methodology (e.g., since 1987, www.nass.usda.gov). We found that the average honey produced per colony per year in the US was positively correlated with both published yearly average (*r* = 0.764, 95%CI = 0.474, 0.904, *p* < 0.0001, n = 19) and model estimated (*r* = 0.502, 95%CI = 0.198,0.719, *p* < 0.0024, n = 34) worker bee median lifespans (Fig. 4). Correlations between honey production and median life span were calculated for survey years 1987–2019, prior to which survey methods differed or were discontinued altogether^24^.

### In-silico population modelling

*As the rate of worker bee mortality increases, BEEHAVE modeling predicts decreases in colony populations, honey yields, and years to mortality (colony longevity)* (Fig. 5). To estimate the potential impacts of documented decreased median life span, we calculated a mortality rate based on the BEEHAVE Systems Model of Colony Dynamics^25^. The daily in-hive mortality rate used by the BEEHAVE model is 0.004, which is calculated from mid-twentieth century observational studies that show the maximal lifespan of overwinter worker bees from healthy colonies is 250 days^20,22,23^. They assume a constant mortality rate over the 250-day time period or 4 in 1000 bees per day, barring exposure to any other risk factors. Applying this thinking to our observations and assuming mean and median lifespan are approximately equal, we estimate that 2010’s Worker Bee Winter Mortality Rate is 0.008,

$$\frac{{1960}^{^{\prime}}s \,Worker\, Bee \,Winter\, Mortality\, Rate}{{1960}^{^{\prime}}s \,Active\, Season\, Worker\, Bee\, Mortality\, Rate}=\frac{{2010}^{^{\prime}}s \,Worker \,Bee \,Winter\, Mortality \,Rate}{{2010}^{^{\prime}}s \,Active\, Season \,Worker\, Bee \,Mortality\, Rate}$$

where active season bees in the 1960’s had a mortality rate of 0.015 (0.5 divided by an average lifespan of 32.5 days), while the mortality rate of the past decade is observed to be 0.030 (0.5 divided by an average median lifespan of 17.7 days).

We compared predicted measures of colony outcomes by adjusting the MORTALITY_INHIVE parameter (either at 0.004 or 0.008) of the BEEHAVE model, which governs the “daily mortality rate of healthy in-hive bees and foragers.” We modeled each mortality rate 1000 times over periods of 10 years. To account for different levels of colony stress, we also ran each modeled mortality rate both with and without treatment for the parasite *Varroa*, for a total of 4 parameter combinations. As each replicate represented a 10-year potential lifespan of 1 colony, the resulting data set represented monthly colony health outcomes for 4000 colonies.

Comparing the two mortality rates as a parameter in the BEEHAVE model, we found that the decreased median life spans observed in the 2010’s predict a 26% decrease in honey production and a 29% decrease in maximum population size, on average. Model replicates using the 1960’s longevity data predicted no colony mortality over 10 years if external threats like *Varroa* were perfectly controlled. By introducing *Varroa* as a problem, the same mortality rate predicts colonies would live an average of 4.6 years. This average decreases by 33%, to 3.1 years when worker bee mortality rates are increased to current estimates and *Varroa* is imperfectly controlled (Fig. 5).

To further understand the implications of these findings, we modeled bee mortality rates adjusting the rate of mortality in increments of 10 pp steps. Each of these models was run 100 times and assumed imperfect *Varroa* control. For each modeled mortality rate, we calculated worker bee lifespan (for May, August, and December), colony lifespans (mean, median, and maximum), annual operational loss rates (with or without replacement), mean maximum population per year, and mean maximum honey production per year (Supplementary Table S1).

For cohorts of colonies that achieved 100% loss in under 10 years, we calculated the average annual loss rate and modeled what would happen within an operation, where lost colonies are continuously replaced, as occurs in commercial beekeeping settings^24^. Assuming a constant loss rate with yearly replacement of lost colonies, we found that the predicted loss rate over time increased by 70% on average (Supplementary Fig. S1, Supplementary Table S1). In the highest loss regimes (in-hive bee mortality rates of 0.0068 or more, Supplementary Table S1), cohorts lost 100% of their colonies in 5 years without replacing dead colonies, an annual loss rate of 20% is predicted. When dead colonies are replaced annually, the average annual rate of loss increases to 33%, a figure close to the average winter loss rate reported in the US over the last 14 years (Supplementary Fig. S1).

## Discussion

Cages of honey bees offered different types of water, in addition to 50% sugar solution, lived longer than those offered sugar syrup alone (Figs. 1 and 2). An analysis of data derived from 46 trials across 25 published cage studies performed over the past five decades in the United States demonstrated that time was the major predictor of differences in median lifespans. This suggests the influence of variables outside the scope of this analysis (Fig. 3). The positive relationship between cage population and median lifespan was unexpected, as previous work shows no difference in mortality rates for cages with less than 100 bees and variable effects for cages with greater than or equal to 100 bees^26–28^. This may be an artifact of the data, where experiments in the 1970’s were more likely to use larger populations while also reporting longer lifespans. Associations between changes in median lifespan and other variables for which existing empirical evidence suggests positive associations (e.g., pollen^29,30^ and pollen substitute^31^) were not found, possibly because their use is not standardized (e.g., pollen from different plants).

Standardized protocols are essential for meaningful comparison of cage trial results across studies. These standardizations should minimize the stress of honey bees to ensure that the risk assessment or other experiments are not confounded by other variables or exposures. Our finding that water provisioned bees lived two to five times longer than deprived bees argues for water’s inclusion in standardized trials. The differences in survivorship between bees offered different kinds of water suggest water can also serve as an important source of micronutrients. Honey bees’ need for micronutrients is thought to drive water foraging choices^9,10,32^ and the benefits associated with a polyfloral pollen diet^30,33^. Until we have a better understanding of micronutrients, and in order to keep standards more consistent across research groups, we recommend that future cage trails provision bees with either deionized water or 1% NaCl in deionized water.

Our finding that cages bees live longer when offered water, both with and without salts, may have important implications for interpreting the results of any cage experiment, where water provision was not part of the experimental protocol. This includes cage experiments done as part of pesticide risk assessments^34–36^, etiological studies (e.g. bees exposed to viruses^37,38^, *Nosema*^39^, *Varroa*^40^), and/or other risk factor (e.g., nutrition^41^ and environment^42^). As all water treatments in our study showed benefit as compared to non-water provisioned controls, water provisioning seems critical to bee health. Certainly, more work is needed to uncouple the effects of water stress on bee physiology including immunity, the detoxification processes, and how water stress may synergize the effects of other stresses (e.g., exposure to pesticides).

Cage trials assessing survival risk are critical because worker bee lifespan has important implications for colony survival. The overall impacts of shorter-lived bees on colony performance likely compounds over time and can go unnoticed until appreciable population declines occur at the end of the season (Fig. 5, Supplementary Fig. S1 and Supplementary Table S1). We document a 50% reduction in worker bee median lifespan that predicts a 33% mean winter loss rate, a rate slightly higher than the average winter loss rates reported by beekeepers over the past 14 years^1^.

Many exposures reduce bee lifespan, and some may explain the reduced longevity we report over the last ½ century. One key factor maybe the establishment and spread of *Varroa* and the viruses it vectors, including Deformed Wing Virus (DWV)^43^, for which some strains have developed increased virulence^44^. Both the parasite and the vectored viruses are known to increases adult bee mortality, accelerate aging, and reduce forager productivity^45,46^. The relationship between the age of bees and colony mortality becomes more apparent when colonies are experiencing stress. Exposures that reduce immunocompetence, such as pesticides^47^ or poor nutrition^48^, either effect bee lifespan directly or can synergize with other factors such as viral infections^49^, causing colony level population dwindling and increased overwinter mortality^50,51^. It is likely that viruses are present in cage trial bee populations. This may explain some of the decreased median lifespans documented by our literature analysis, especially since the mid-1980’s when *Varroa* were first introduced^52^. The near universal presence of DWV in US bee populations makes the option of using disease free specimen in cage trails unworkable, and so we recommend that all cage trials report the viral presence and load of their source colonies. This then will allow for the consideration of viral effects when interpretating results. Along these lines, we would also recommend analyzing the wax and bee bread from bee source colonies for the presence of pesticides, as exposure to such stressors at the larval stage may affect adult lifespan^53^.

Bee longevity, like any phenotypic expression, is the result of both environmental exposure and the bees’ genome. Most measurements that compare bees of various ages, from the tissue to the molecular level, point to associations with task performance, rather than chronological age^54^. Work that considers task plasticity or task stagnation as part of the experimental design have revealed associations between chronological aging and proteomic changes^55^, mandibular gland development^56^, and the accumulation of lipid-protein aggregates^57^. Genetic mechanisms that track with age could reveal heritable variability among honey bee populations. Increasing longevity is possible to some extent through selective breeding^58,59^. In truth, the effect that “long-lived bees” would have on colony health is largely unknown^13^, although the general assumption is that longer lived bees are associated with larger populations and greater honey production. Intriguingly, pathogens and parasites that affect adult honey bees are less immediately destructive as compared to diseases of the brood^60^. Pathogens that do not kill adult bees outright are able to accumulate within the population, so colonies with shorter lived bees would have reduced pathogen and disease loads when compared to those with longer lived bees. In this scenario, colonies with shorter lived bees would appear healthier and would be favored by breeders, who may be inadvertently selecting for reduced lifespans in adult bees.

Regardless of the cause(s), shortened worker bee lifespans have predictable implications for colony health and survivorship. Our modeling of honey bee populations shows direct relationships between adult worker bee mortality rates, population size, and honey production. Field studies show that honey produced per colony is correlated to the amount of brood^61^, queen age^62^, population size^63^, as well as worker bee lifespan^13^. This effect is confirmed by several other population models where colonies with shorter-lived bees displayed increases in precocious foraging, disruptions to colony age structure, and higher overall mortality^15,17–20^. Here we show a strong relationship between reported (r = 0.76) or model estimated (r = 0.50) bee median lifespans and the average honey produced per colony over the same time period. These results indirectly support our concluding hypothesis that bees have suffered decreased lifespan over the last 50 years.

The reduction in lifespan observed in our literature analysis could underpin many of the frustrations that US beekeepers report. For instance, reduced queen lifespan is reported consistently as a problem for commercial beekeepers^1^. It is unknown, but seems feasible, that worker and queen lifespan are linked. Queen lifespans are highly variable, as they are affected by a host of exposures, however, there is a prominent difference in queen age reported over time. In the 1960’s, queens with an average lifespan of 5 years were reported, while after 1978, queen lifespans beyond 1–3 years were no longer reported. The introduction of *Varroa*, changes in virulence of associated bee viruses in the late 1980’s, and the products used to control Varroa are estimated to account for 50% of the reduction in queen lifespan^64^. Still, no work has explored the longevity of queens between the various bee lines.

The centrality of worker bee lifespan to colony health suggests an urgent need to better understand factors that drive it. Given that honey bees display genetic variation to longevity^65^ and that length of life is a heritable trait^58,59^, we should be able to determine the effect of increasing worker bee lifespans on the health of entire colonies. Currently we concentrate our efforts on reducing colony stress, like supplementary feeding or reducing parasitic pressure^66^. Changes in worker bee lifespan and approaching water as a beneficial metabolite adds to the existing framework for understanding how multiple stressors can increase colony loss rates.

The paucity of data in published literature did not permit looking at lifespan over time in other regions than the US. Should this phenomenon have a geographic component, it would easily direct the formation of hypotheses that may reveal the underlying causes. Understanding the relationships between reduced lifespans and colony loss rates would be equally important. To do so would require collaboration among international teams of researchers able to recreate work from the 1960’s that measured seasonal longevity, while screening queen breeders for associations with heritable variation.

## Materials and methods

### Cage experiment

Honey bees were obtained from five source colonies located at the University of Maryland, College Park campus. Colonies were inspected bimonthly throughout the active season and *Varroa* treatments were applied as needed to keep below a threshold of one mite per one hundred bees. Brood frames from each colony were harvested in September 2017 and kept in an incubator at 32 °C 65% relative humidity. Newly emerged bees were removed from brood frames in under 24 h and transferred to cages. We executed a nested block design, where each block consisted of three treatment groups and one control group. There was a total of five blocks, with each containing bees from the same source colony. All cages received a diet of 50% sucrose solution and the pollen substitute *MegaBee* (megabee.com) through modified 2 ml microcentrifuge tubes. In addition to this diet, treatment groups were offered either a 1% NaCl solution in deionized water, deionized water, or tap water. Selection of treatments was based on ease of accessibility or standardization. All feeders were weighed and replaced daily. Dead bees were counted and removed daily. The experiment was scheduled to run for a minimum of three weeks or until the control cages reached their median lifespans.

### Literature analysis

Two separate literature searches were performed on February 21st, 2020, and April 28th, 2020, using Web of Science and Google Scholar. Search parameters included (“honey bee” or “honeybee” or “*Apis mellifera*”) and (“cage” or “incubator” or “cage trial” or “laboratory cage trial” or “median lifespan” or “survivorship” or “lifespan” or “longevity”). A total of 111 publications from 1970 to 2019 were initially identified as incubator cage trials of honey bees. From these, data was extracted on the year of publication, dependent and independent variables, cage population, the presence or absence of dietary variables (water, sucrose, pollen, pollen substitute, and honey), the source or type of water, pollen, or pollen substitute offered, sucrose type and concentration, the presence of a buffer in any fluids offered, the use of commercial Queen Mandibular Pheromone (QMP), incubator temperature and humidity, the age of the bees at the beginning of the experiment, median lifespans of control groups, duration of the experiment, the season the bees were harvested, and the country and state/province/territory hosting the experiment.

Criterion for inclusion in the regression was limited to studies that reported median lifespan as an experimental outcome, newly emerged bees as the source specimen, and experimental design variables related to cage environment. Studies from countries outside the US were removed due to the lack of consistency of data reported over the designated period (Australia n = 1, Germany n = 1, Canada n = 3, England n = 1, France n = 4, Italy n = 1, New Zealand n = 6, Poland n = 1, Saudi Arabia n = 1, Thailand n = 1, Taiwan n = 1, South Africa n = 1). Infrequently reported variables or those with limited variation were also excluded from the analysis (honey n = 2, season of specimen collection n = 8, use of dietary buffer n = 1, commercial QMP n = 1, water source n = 5). This resulted in 46 trials over 25 studies on which to perform a meaningful regression.

### Honey production

The Quick Stats database of the USDA National Agricultural Statistics Service (www.nass.usda.gov) was accessed on March 25th, 2021. Data export represented the average annual honey production at the national level for 1986–2019. The survey was discontinued between 1982 and 1985. Honey production figures prior to 1982 were acquired from the USDA Economics, Statistics, and Marketing Information System (usda.library.cornell.edu) but were excluded from the analysis due to differences in survey methodology. These data were then combined with either the observed median lifespans recorded in the literature analysis, or the median lifespans predicted by the literature analysis regression.

### Population modeling

Two experiments were conducted for the theoretical effect of varying mortality rates on colony health and productivity using the BEEHAVE Systems Model of Honeybee Colony Dynamics (https://beehave-model.net)^25^. The first experiment compared model outputs for honey production, colony population, and colony longevity between colonies experiencing different mortality rates of in-hive bees and measures of *Varroa* control, a total of 4 distinct colony groups. The model parameter MORTALITY_INHIVE was either left to the default setting (0.004) or adjusted to a rate derived from median lifespans observed in the Literature Analysis from the past decade (0.008, see results). Under each mortality setting, data was collected for colonies experiencing either perfect (treatment applied) or imperfect control (treatment not applied) of *Varroa* populations. Data was recorded daily over a 10-year period for each colony and was replicated 1000 times using Python 3.7^67,68^ and the *PyNetLogo* package^69^. For each replicate, data was summarized to the mean maximum honey produced, mean maximum population size, and the length of colony life.

The second experiment left *Varroa* untreated while increasing the mortality rate by 10 percentage points until reaching a 100% increase, representing 10 years of data for 11 groups of colonies over 100 replicates each. The same metrics from the first experiment were collected with the addition of the average lifespan for in-hive bees in May, August, and December. For each group of colonies, 100% loss was divided by the total number of years alive to calculate an annual loss rate without replacement. These annual loss rates were then extrapolated to the operational level, where lost colonies are continuously replaced, and applied to each new cohort of colonies added over time.

### Statistical analysis

All statistical analysis was conducted in R^70^ and figures were produced using the packages *ggplot2*^71^ and *survminer*^72^.

For the Cage Experiment, Kaplan–Meier survivorship curves^73^ were generated using the *survival* package^74^. Cox Proportional Hazards with Mixed Effects were calculated using the *coxme* package^75^. Model selection was based on AICc criterion^76^ between the full model with random effects (cage nested in block), the full model without random effects, and the null model with random effects. For the Literature Analysis, comparisons were made between changes in median lifespan and experimental design variables (cage population, water use, pollen use, pollen substitute use), as well as across time. In order to eliminate local effect sizes of modeled variables, only the most basic, within-study control group experimental designs were included in the regression. Models were generated with the *lmerTest* package^77^ and random effects structures were compared between source publication, U.S. State where specimen were reared, and the climate zone where specimen were reared. No significant differences were detected in model fit between the three random effect structures, so U.S. State was used as it represents the highest resolution grouping factor with the fewest number of single within-group observations (9 out of 46 trials). Model selection was exploratory using the *MuMIn* package^78^ and based on AICc criterion. Two models were produced through this exploration and the simpler of the two was chosen as the final model. For Honey Production figures, normality of data was confirmed using the Shapiro–Wilk Normality Test and Pearson’s Correlations were generated between average annual honey production and either actual or model predicted median lifespans. For the Population Models, pairwise comparisons were performed using Wilcoxon Rank Sum test with the Benjamini–Hochberg procedure for controlling false discovery rate.

## Supplementary Information

Supplementary Information.
