# Supplementary material for: Water provisioning increases caged worker bee lifespan and caged worker bees are living half as long as observed 50 years ago
Source: Sci Rep. 2022 Nov 14;12:18660. doi: 10.1038/s41598-022-21401-2 (PMC9663547; doi:10.1038/s41598-022-21401-2)
Supplement: Supplementary file 1 — Supplementary Information. [file 41598_2022_21401_MOESM1_ESM.pdf]

	Worker Bee (predicted)			Colony (predicted)						
BEEHAVE Mortality Rate	Lifespan (days, Mean (SE))			Lifespan (years)			Annual Loss Rate		Maximum Population	Annual Honey Production (kg)
	May	August	December	Mean (SE)	Median	Max	Without Replacement	With Replacement	Mean (SE)	Mean (SE)
0.0040*	62.54 (0.67)	24.96 (0.07)	59.36 (0.53)	4.67 (0.06)	4.21	7	14%	25%	37323 (160)	48.30 (0.18)
0.0044	61.49 (0.68)	24.72 (0.07)	59.39 (0.54)	4.44 (0.06)	3.9	7	14%	25%	36267 (128)	47.47 (0.18)
0.0048	58.78 (0.69)	24.44 (0.07)	60.68 (0.56)	4.10 (0.04)	3.55	6	17%	28%	35230 (152)	46.34 (0.23)
0.0052	58.94 (0.70)	24.09 (0.07)	58.69 (0.55)	4.13 (0.05)	3.57	6	17%	28%	34384 (121)	45.30 (0.20)
0.0056	55.67 (0.68)	23.75 (0.07)	59.28 (0.55)	4.06 (0.05)	3.55	6	17%	28%	33294 (132)	43.69 (0.22)
0.0060	55.42 (0.69)	23.62 (0.07)	59.66 (0.56)	3.86 (0.05)	3.4	6	17%	28%	32382 (153)	42.51 (0.24)
0.0064	54.48 (0.69)	23.37 (0.07)	58.25 (0.57)	3.77 (0.05)	3.34	6	17%	28%	31474 (156)	41.28 (0.26)
0.0068	52.21 (0.70)	22.98 (0.07)	58.47 (0.58)	3.54 (0.05)	3.06	5	20%	33%	30425 (192)	39.59 (0.26)
0.0072	52.37 (0.71)	22.69 (0.07)	58.28 (0.58)	3.42 (0.05)	2.85	5	20%	33%	29761 (176)	39.26 (0.27)
0.0076	50.51 (0.71)	22.30 (0.07)	56.43 (0.59)	3.29 (0.05)	2.71	5	20%	33%	29139 (210)	37.82 (0.33)
0.0080**	48.93 (0.71)	21.95 (0.07)	56.58 (0.60)	3.21 (0.08)	2.6	5	20%	33%	28236 (221)	36.74 (0.31)

Supplementary Table S1 – BEEHAVE modelling outputs after 100 replicates for each change in mortality rate increase, where Varroa were left untreated and a 10pp increase in mortality rate of worker bees in modeled colonies. Outputs for each step towards a doubling of mortality rate reports the average worker lifespan (longevity), the average lifespan of a colony (longevity), the median lifespan of a colony, the maximum colony lifespan, the average loss rate per year without replacement of dead colonies and the average loss over time when lost colonies are replaced (Fig 6), the average maximum colony population over the year, and the average maximum amount of honey production per colony per year in kg.
*Mortality rate estimated from mid-20th century observational data.
**Mortality rate estimated from caged bee median lifespans 2010-2019.

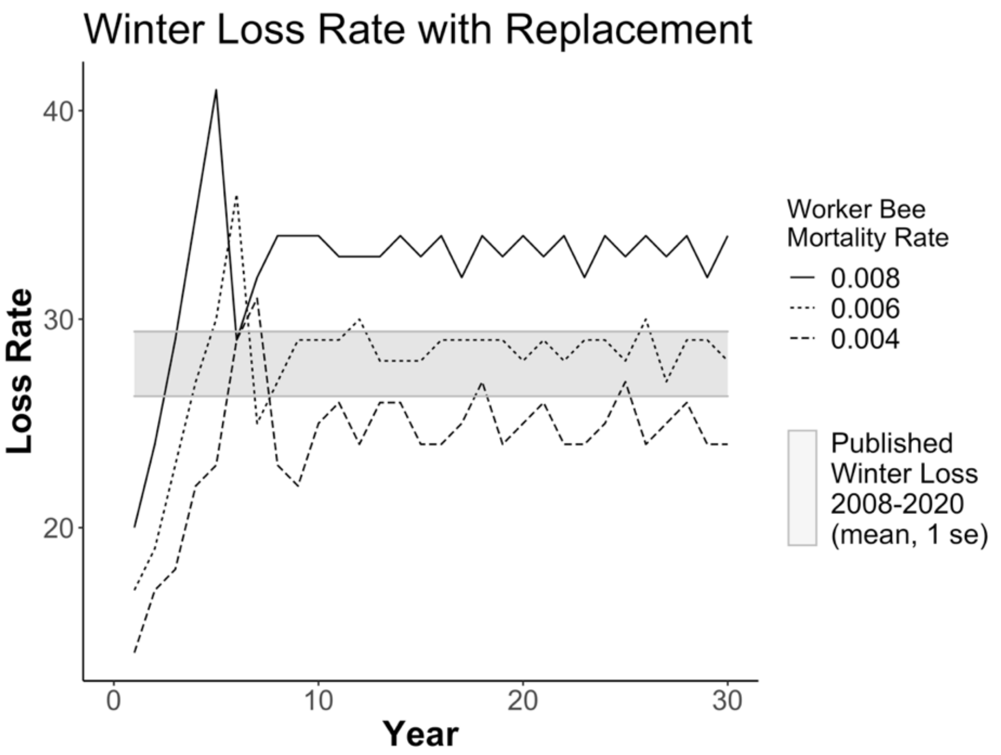

Supplementary Figure S1 – Predicted operational loss rate over time, assuming annual replacement of dead colonies, when worker bees are assumed to live an average of 32.5 days (daily mortality rate = 0.004), 24.9 days (daily mortality rate = 0.006), and 17.7 days (daily mortality rate = 0.008). Mean and one standard error for National US Winter Loss Rates range (27.6% +/- 1.5%, shaded grey) corresponds to an average worker bee life span of 24.5 days. 100% loss was divided over the number of years a cohort survived without replacement. We assume each new cohort of colonies added to replace those lost the year prior experienced the same constant loss rate as those surviving the previous year.

Reference	Modeled	#Trials	Notes
Haydak, M. & Levin, M. Antibiotics and the longevity of newly emerged honey bees. *Annals of the Entomological Society of America* **63**, 344-346 (1970).	y	2	
Knox, D. A., Shimanuki, H. & Herbert, E. Diet and the longevity of adult honey bees. *Journal of Economic Entomology* **64**, 1415-1416 (1971).	y	2	
Moffett, J. O., Morton, H. L. & MacDonald, R. H. Toxicity of some herbicidal sprays to honey bees. *Journal of Economic Entomology* **65**, 32-36 (1972).	n	1	Median lifespan not reported
Morton, H. L., Moffett, J. O. & Macdonald, R. H. Toxicity of herbicides to newly emerged honey bees. *Environmental Entomology* **1**, 102-104 (1972).	y	14	
Kulincevic, J. M., Rothenbuhler, W. C. & Stairs, G. R. The effect of presence of a queen upon outbreak of a hairless-black syndrome in the honey bee. *Journal of Invertebrate Pathology* **21**, 241-247 (1973).	n	1	Not all caged bees were newly emerged
Lackett, J. J., Burkhardt, C. & Hitchcock, J. Oxodene: longevity of honey bees. *Journal of Economic Entomology* **65**, 19-20 (1972).	y	1	

Rinderer, T. E. & Dell Elliott, K. Worker honey bee response to infection with Nosema apis: influence of diet. *Journal of Economic Entomology* **70**, 431-433 (1977).	y	2	
Rinderer, T. E. & Sylvester, H. A. Variation in response to Nosema apis, longevity, and hoarding behavior in a free-mating population of the honey bee. *Annals of the Entomological Society of America* **71**, 372-374 (1978).	y	2	
Rinderer, T. E. & Baxter, J. R. Honey bees: the effect of group size on longevity and hoarding in laboratory cages. *Annals of the Entomological Society of America* **71**, 732-732 (1978).	y	1	
Westerdahl, B. B. & Gary, N. E. Longevity and food consumption of microwave-treated (2.45 GHz Cw) honeybees in the laboratory. *Bioelectromagnetics: Journal of the Bioelectromagnetics Society, The Society for Physical Regulation in Biology and Medicine, The European Bioelectromagnetics Association* **2**, 305-314 (1981).	y	1	
Johansen, C. A., Mayer, D. F., Eves, J. D. & Kious, C. W. Pesticides and bees. *Environmental Entomology* **12**, 1513-1518 (1983).	n	1	Median lifespan not reported
Rinderer, T. E., Collins, A. M. & Brown, M. A. Heritabilities and correlations of the honey bee: Response to Nosema apis, longevity, and alarm response to isopentyl acetate. *Apidologie* **14**, 79-85 (1983).	n	1	Median lifespan not reported
Kulinčević, J. M. & Rothenbuhler, W. C. Selection for length of life in the honeybee (Apis mellifera). *Apidologie* **13**, 347-352 (1982).	y	1	

Mayer, D. & Lunden, J. Toxicity of fungicides and an acaricide to honey bees (Hymenoptera: Apidae) and their effects on bee foraging behavior and pollen viability on blooming apples and pears. *Environmental Entomology* **15**, 1047-1049 (1986).	n	1	Median lifespan not reported
Vandenberg, J. D., Hamm, J. J. & Shimanuki, H. Vairimorpha sp. spores do not reduce the longevity of caged adult honey bees, Apis mellifera (Hymenoptera: Apidae). *Environmental Entomology* **15**, 207-209 (1986).	y	2	
Schmidt, J. O., Thoenes, S. C. & Levin, M. D. Survival of honey bees, Apis mellifera (Hymenoptera: Apidae), fed various pollen sources. *Annals of the Entomological Society of America* **80**, 176-183 (1987).	y	3	
Eischen, F. A., Vergara, C., Dietz, A. & Cardoso-Tamez, D. Cymiazole, a systemic acaricide that controls Acarapis woodi (Rennie) infesting honey bees. I. Laboratory tests. *Apidologie* **19**, 367-376 (1988).	y	1	
Vandenbergi, J. Safety of four entomopathogens for caged adult honey bees (Hymenoptera: Apidae). *Journal of Economic Entomology* **83**, 755-759 (1990).	y	2	
Webster, T. C. Fumagillin affects Nosema apis and honey bees (Hymonopterai Apidae). *Journal of Economic Entomology* **87**, 601-604 (1994).	n	1	Median lifespan not reported; Cage population not reported
Alves, S., Marchini, L., Pereira, R. & Baumgratz, L. Effects of some insect pathogens on the Africanized honey bee, Apis mellifera L.(Hym., Apidae). *J. Appl. Entomol.* **120**, 559-564 (1996).	y	1	

Iwasa, T., Motoyama, N., Ambrose, J. T. & Roe, R. M. Mechanism for the differential toxicity of neonicotinoid insecticides in the honey bee, Apis mellifera. *Crop Protection* **23**, 371-378 (2004).	n	1	Median lifespan not reported
Yang, X. L. & Cox-Foster, D. L. Impact of an ectoparasite on the immunity and pathology of an invertebrate: evidence for host immunosuppression and viral amplification. *Proc. Natl. Acad. Sci. U. S. A.* **102**, 7470-7475 (2005).	y	1	
Rose, R., Dively, G. P. & Pettis, J. Effects of Bt corn pollen on honey bees: emphasis on protocol development. *Apidologie* **38**, 368-377 (2007).	y	1	
Bromenshenk, J. J. *et al.* Iridovirus and Microsporidian Linked to Honey Bee Colony Decline. *PloS ONE* **5**, e13181 (2010).	y	1	
Wu, J. Y., Anelli, C. M. & Sheppard, W. S. Sub-lethal effects of pesticide residues in brood comb on worker honey bee (Apis mellifera) development and longevity. *PloS one* **6**, e14720 (2011).	n	1	Cage population not reported
Milbrath, M. O., Xie, X. & Huang, Z. Y. Nosema ceranae induced mortality in honey bees (Apis mellifera) depends on infection methods. *Journal of invertebrate pathology* **114**, 42-44 (2013).	y	1	
Goblirsch, M., Huang, Z. Y. & Spivak, M. Physiological and Behavioral Changes in Honey Bees (Apis mellifera) Induced by Nosema ceranae Infection. *PLoS ONE* **8**, e58165, doi:10.1371/journal.pone.0058165 (2013).	y	1	

Berry, J. A., Hood, W. M., Pietravalle, S. & Delaplane, K. S. Field-level sublethal effects of approved bee hive chemicals on honey bees (Apis mellifera L). *PloS one* **8**, e76536 (2013).	y	1	
Huang, S. K. *et al.* Evaluation of cage designs and feeding regimes for honey bee (Hymenoptera: Apidae) laboratory experiments. *Journal of economic entomology* **107**, 54-62 (2014).	y	1	
Milbrath, M. O. *et al.* Comparative virulence and competition between Nosema apis and Nosema ceranae in honey bees (Apis mellifera). *Journal of invertebrate pathology* **125**, 9-15 (2015).	y	1	
Dolezal, A. G., Carrillo-Tripp, J., Miller, W. A., Bonning, B. C. & Toth, A. L. Pollen contaminated with field-relevant levels of cyhalothrin affects honey bee survival, nutritional physiology, and pollen consumption behavior. *Journal of economic entomology* **109**, 41-48 (2016).	y	1	
Liao, L.-H., Wu, W.-Y. & Berenbaum, M. R. Impacts of dietary phytochemicals in the presence and absence of pesticides on longevity of honey bees (Apis mellifera). *Insects* **8**, 22 (2017).	y	1	
Gregorc, A. *et al.* Effects of coumaphos and imidacloprid on honey bee (Hymenoptera: Apidae) lifespan and antioxidant gene regulations in laboratory experiments. *Sci Rep* **8**, 1-13 (2018).	n	1	Median lifespan not reported
Li, J. *et al.* Pollen reverses decreased lifespan, altered nutritional metabolism and suppressed immunity in honey bees (Apis mellifera) treated with antibiotics. *Journal of Experimental Biology* **222** (2019).	y	1	

Supplementary Table S2 - Publications in the US assessed for inclusion of control group data in linear modeling. See Methods section for detailed description of inclusion criteria.
